# WHO’S UP NEXT? EXTENDED FAMILY CAREGIVERS OF PERSONS LIVING WITH DEMENTIA: CURRENT AND FUTURE CARE

**DOI:** 10.1093/geroni/igad104.0056

**Published:** 2023-12-21

**Authors:** Tina Savla, Karen Roberto

**Affiliations:** Virginia Tech, Blacksburg, Virginia, United States; Virginia Polytechnic Institute & State University, Blacksburg, Virginia, United States

## Abstract

Various societal trends and expanded family boundaries have led to a broad array of family members involved in caring for persons living with dementia (PLwD). We examined how extended family caregivers (FCG) manage their care responsibilities and how the care arrangement impacts the provision of future care for PLwD. Mixed-method data from 48 extended FCG and 56 nuclear FCG (71% White, 68% female, 63% co-reside with PLwD) revealed that 70% of extended FCG rely on informal helpers to care for PLwD. Spouses and daughters were more often sole caregivers while most sons relied on informal helpers. If no longer able to help, sisters, granddaughters, and nieces more likely foresaw moving PLwD to a care facility, whereas brothers, grandsons, and nephews anticipated another informal helper would assume their care. Extended FCG often expressed dissatisfaction with one or more informal helpers for failing to meet care responsibilities, potentially leading to unmet needs and increased risk for early nursing home placement for the PLwD. The discussion will focus on the attributes of informal helpers of extended FCG that contribute to stable or inconsistent care arrangements for PLwD, which can ultimately affect the well-being of PLwD and FCG.

